# *t*-LSE: A Novel Robust Geometric Approach for Modeling Protein-Protein Interaction Networks

**DOI:** 10.1371/journal.pone.0058368

**Published:** 2013-04-01

**Authors:** Lin Zhu, Zhu-Hong You, De-Shuang Huang, Bing Wang

**Affiliations:** 1 Intelligent Computing Lab, Institute of Intelligent Machines, Chinese Academy of Sciences, Hefei, Anhui, China; 2 Department of Automation, University of Science and Technology of China, Hefei, Anhui, China; 3 College of Computer Science and Software Engineering, Shenzhen University, Shenzhen, Guangdong, China; 4 School of Electronics and Information Engineering, Tongji University, Shanghai, China; University Of Oxford, United Kingdom

## Abstract

Protein-protein interaction (PPI) networks provide insights into understanding of biological processes, function and the underlying complex evolutionary mechanisms of the cell. Modeling PPI network is an important and fundamental problem in system biology, where it is still of major concern to find a better fitting model that requires less structural assumptions and is more robust against the large fraction of noisy PPIs. In this paper, we propose a new approach called *t*-logistic semantic embedding (*t*-LSE) to model PPI networks. *t*-LSE tries to adaptively learn a metric embedding under the simple geometric assumption of PPI networks, and a non-convex cost function was adopted to deal with the noise in PPI networks. The experimental results show the superiority of the fit of *t*-LSE over other network models to PPI data. Furthermore, the robust loss function adopted here leads to big improvements for dealing with the noise in PPI network. The proposed model could thus facilitate further graph-based studies of PPIs and may help infer the hidden underlying biological knowledge. The Matlab code implementing the proposed method is freely available from the web site: http://home.ustc.edu.cn/~yzh33108/PPIModel.htm.

## Introduction

Proteins are crucial for almost all of functions in the cell. Usually, they rarely perform their functions alone, but cooperate with each other by forming a huge network of protein-protein interactions (PPIs). In the past decades, many innovative techniques for detecting PPIs have been developed [1]–[4]. Benefited from the progress in large-scale experimental technologies such as yeast two-hybrid (Y2H) screens [1], [5], tandem affinity purification (TAP) [2] and mass spectrometric protein complex identification (MS-PCI) [3], a large amount of PPI data for different species has been accumulated [1]–[3], [5]–[7]. PPI data are usually represented in term of graph (network), where nodes in the graph represent proteins, and there is an edge between two nodes if the corresponding proteins interact with each other. PPI networks provide a comprehensive view of the global interaction structure of an organism's proteome, as well as detailed information on specific interactions [8]. Analyzing its structure could lead to new knowledge about complex biological mechanisms.

To analyze the PPI networks, an important step is to find an adequate model which could generate networks that closely replicate the structure of real PPI data [9]. It could give insights into understanding and replicating the biological processes and the underlying complex evolutionary mechanisms that created the networks [10], [11], it will also be helpful for understanding biological function, disease and cell's evolution.

On the other hand, up to now there is no complete and highly reliable PPI network of any organism available. Even the most studied PPI network of *yeast* is very noisy and far from being complete [12]–[14]. A good PPI network model can be used as a convenient mathematical framework for dealing with one of the biggest challenges in PPI networks research: detection of huge levels of false positives and false negatives protein interactions [15].

In addition, due to the NP-hard nature of many global systems biology problems, most of graph-theoretic approaches have been proven to be computationally infeasible for biological network analysis in comprehensive genome-scale. However, special classes of graphs usually have given network properties, which makes settling many problems on such graph classes practicable. Therefore, modeling PPI networks by some special graph classes could simplify the computational manipulation and make it easier to extract biological knowledge which is encoded in the network structure. Furthermore, a well-fitting network model can be used to guide biological experiments in a cost and time optimal way. For example, Lapp et al. used the scale-free model of human PPI network to optimize their biological experiments, by which up to 90% of the human interactome can be detected with less than one-third of the proteome used as bait in large-scale pull down experiments [16].

Currently, many network models have been proposed to describe PPI networks. The very first attempts began with Erdos-Renyi(ER) random graphs, the earliest network model [17]. In a random graph with *n* vertices, each of the possible edges between pairs of vertices are distributed uniformly at random with same probability *p*, which means that all vertices have nearly the same degree, i.e. the probability of two vertices to interact equals *p* in ER model. The clustering coefficient of two vertices also equals to *p* which is much smaller than that in many real PPI networks. Therefore, the ER model fails to reproduce even the simplest network properties of PPI networks. Other better fitting models for PPI network, therefore, were introduced recently. In generalized random graphs (ER-DD), the edges are randomly chosen as in ER graphs, but the degree distribution is constrained to match the degree distribution of the real networks [18]. Small world (SW) networks are characterized by small diameters and large clustering coefficients [19]. Modeling the PPI network data by scale-free (SF) network, a network whose degree distribution follows a power-law, is based on the assumption that the degree distribution is one of the most important network properties that a network model should capture [20]. However, it has been shown that two networks with exactly the same degree distribution can have completely different network structures [9]. Higham et al. propose to model PPI networks based on stickiness index [21], where vertices with high stickiness index mimic proteins with many complimentary physical aspects. It is shown that fitting a stickiness model can produce better results than simply choosing a degree-matching graph uniformly at random.

The above-mentioned models were introduced to capture specific network properties or mimic the way that the networks might be evolved. However, they do not utilize the connectivity information of the PPI networks to learn the networks topological structures. Przulj et al. proposed a new model [15], [22] which can exploit the entire connectivity information of a PPI network to learn its structure. Their approach is based on the geometric assumption of PPI networks, i.e., in a PPI network nodes correspond to points in a metric space and edges are created between pairs of nodes if the corresponding points are close enough in the metric space according to some distance norm [23], [24]. The geometric assumption is justified by the demonstration that PPI networks can be explicitly embedded into a low-dimensional geometric space [15], [23], [25]. On the other hand, it has been approved that all biological entities, including genes and proteins as gene products, exist in some multidimensional(likely metric) biochemical space. It is also likely to include as dimensions phenomena such as post-translational modifications, small molecule bindings, etc. Mathematically, we can consider these properties to be dimensions of some abstract metric space [11].

Given the connectivity matrix of a PPI network, Przulj's model (denoted as MDS-GEO henceforth) firstly constructs a distance matrix between the proteins which satisfy the geometric assumptions. Then the proteins are embedded into a low-dimensional space using multidimensional scaling (MDS), i.e., the spectral decomposition of the distance matrix [15]. Experiments show that they achieved a substantial improvement in the fit of their model to PPI networks over all other currently commonly used random graph models [25].

MDS-GEO has also been successfully applied to identify the false positive links in the PPI networks: after the embedding is learned, a pair of proteins that is connected in the original PPI network will be assigned an interaction if and only if they are close to each other in the embedded space. Although only the topological information of PPI networks was utilized in MDS-GEO, its overall performance is competitive with that of biological experimental techniques and methods that combine additional information [22].

Despite the advantages of MDS-GEO model, its performance is limited by some drawbacks: (1) MDS-GEO seeks to preserve a predefined metric. Obviously, beside the geometric assumption, MDS-GEO enforces more structural assumptions on the embedding and may deteriorate the fitting performance. (2) The PPI networks are known to contain a lot of noise [22]. However this problem is not well addressed in MDS based methods [12]. Equipped with the 2-norm cost function, MDS is known to be sensitive to outliers [26]. Furthermore, MDS-GEO uses the shortest path-lengths on the graph to define the similarity between nodes, which is also sensitive to the false-positive links in the graph [27].

In this paper, we propose a novel approach, *t*-logistic semantic embedding (*t*-LSE), to model PPI networks. Like in MDS-GEO, our approach is also based on the geometric assumption and requires only the connectivity information of the PPI network. However, *t*-LSE does not seek to preserve a predefined metric. Instead, we adaptively learn a metric embedding under the criterion that it can better satisfy the geometric assumption. Under this flexible learning framework, the experimental results show that *t*-LSE can embed PPI network into low dimensional metric space more successfully than MDS-GEO in terms of various evaluation metrics.

On the other hand, inspired by recent work in machine learning domains like robust classification [28], [29], we adopt a non-convex cost function to deal with the noise in PPI networks. To the best of our knowledge, this is the first work that uses this technology to learn robust graph embedding from noisy connectivity information. The experimental results show that *t*-LSE can identify the topology of the original PPI network under various levels of random perturbation. Moreover, it is further successfully applied to assess false-positive PPI links. The experimental results demonstrated the present method can achieve a big performance improvement in dealing with the noise in PPI network.

## Results and Discussion

### Data Sources and Evaluation Metric

In this work, physical PPI networks of three eukaryotic organisms: human Homo sapiens, yeast Saccharomyces cerevisiae, and fruitfly Drosophila melanogaster are analyzed. There are a total of 5 PPI networks, three of which are human, one is yeast, and one is fruitfly.

We denote by H_InAct, H_Bind, H_BioGrid the human PPI networks from curated databases IntAct [30], BIND [31], and BioGrid [32], respectively (They were downloaded on February 10, 2010). Similarly, Y_Tong and F_BioGrid denote the yeast and fruitfly PPI networks from [32], [33]. Thus, we are using PPI networks of different confidence levels that come from a range of high throughput PPI detection technologies as well as from human curation. The characteristics of five protein interaction data are listed in Table 1.

**Table 1:** Characteristics of five protein interaction data.

Networks	Organisms	Number of Nodes	Number of Edges
Y_Tong	Yeast	2171	7622
F_BioGrid	Fruitfly	6675	19970
H_InAct	Human	4486	13807
H_Bind	Human	3276	6474
H_BioGrid	Human	7493	27045

**Figure 1:**
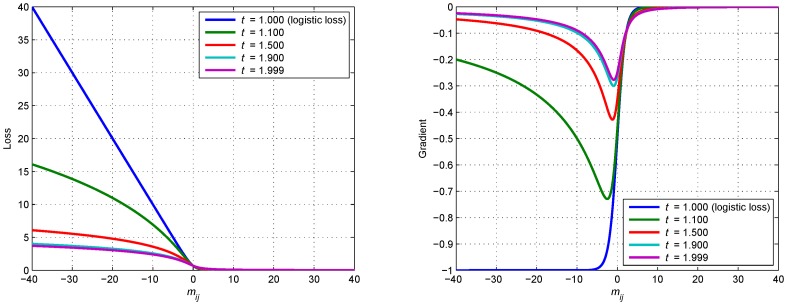
The *t*-logistic loss function. The *t*-logistic loss function (left) and its gradient (right), with *t* = 1, we recover the logistic loss.

As is defined in [28], the parameter *t* of the *t*-logistic loss function should take value between 1 and 2. With *t* close to 1, the *t*-logistic loss function is similar to the convex logistic loss function, since we propose using non-convex loss function in *t*-LSE, we mainly evaluate the results when *t* takes 2 at extreme points. As is illustrated in Figure 1, the difference between *t*-logistic loss function with *t* = 1.9 and *t*-logistic loss function with *t* approaching 2(e.g., *t* = 1.999) is very small, therefore *t* is set to 1.9 during the experiments unless clearly stated.

We adopted three measurements, i.e., graphlet degree distribution (GDD) agreement [25], receiver operator characteristic (ROC) curve and probability density function, to evaluate the performance of *t*-LSE, and then assess its robustness via its ability in identifying false positives in real PPI networks.

### GDD Agreement Comparison of Various Models

A well fitting network model should generate graphs which closely resemble the structure of original PPI networks. To evaluate the fit of proposed network model to PPI data, we should compare the model networks with the original PPI networks. However, direct comparisons of large networks is computationally infeasible due to the NP-completeness of the underlying subgraphs isomorphism problem. Instead, it usually rely on heuristics which are commonly called *network properties* [25]. The heuristics could be divided into two groups: global and local properties. Global properties include different kinds of network centralities, such as degree distribution, clustering coefficient, et al. Local properties include network motifs and graphlets, both of which indicate the occurrence of small subgraphs in a large network. Because current PPI networks are unfortunately incomplete and rife with noise [13], global properties of such dirty data might be biased or even contain misleading information, whereas local properties are likely to be valid and meaningful. On the other hand, cell biology is thought of as modular; many pathways and feedback loops are inherently seen as detachable modules [34]. Although it has been proven that network motifs alone do not determine function in general, there is the possibility of a close connection between subgraphs and biological functionality [35]. Therefore, we employ local network similarity GDD agreement [25], [36] to compare the model network with the original PPI networks.

The GDD agreement is a similarity measure between the GDDs of two networks, where GDD measures the percentage of nodes ‘touching’ a specific number of graphlets. The GDD agreement ranges from 0 to 1. If it is close to 1, it denotes that two networks have similar GDDs, and otherwise, their GDDs are different.

We compare *t*-LSE with five commonly used network models listed in Table 2. The model network generators are implemented as follows: ER graphs are generated by the LEDA random graph generator [37]. ER-DD graphs are generated by using the “stubs method” [38]: the number of “stubs” (to be filled by edges) is assigned to each node in the model network according to the degree distribution of the original PPI network being modeled; edges are created between pairs of nodes picked at random; after an edge is created, the number of “stubs” left available at the corresponding “endnodes” of the edge is decreased by one. SF networks are generated by using the Barabási-Albert preferential attachment model [20]. In our implementation, we use GraphCrunch [24] to calculate the GDD agreement. Each network models matched the number of nodes and edges in the corresponding PPI network.

**Table 2:** Models used to model PPI networks.

Network Model	Reference	Input Information
ER	Erdos-Renyi random graph model [17]	The number of edges and nodes
ER-DD	ER model with the same degree distribution as in original data [18]	The number of edges and nodes and the degree distribution
MDS-GEO	[15]	The connectivity matrix
SF	Scale-free Barabasi-Albert preferential attachment model [20]	The number of edges and nodes and the degree distribution
Sticky	Stickiness-index based model [21]	The number of edges and nodes and the degree of each individual node

**Figure 2:**
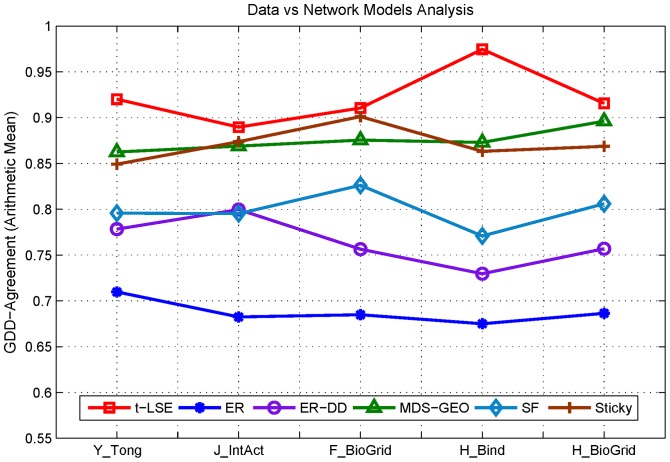
The GDD-agreement between the original PPI networks and the model networks. The horizontal axis denotes five different PPI networks described in Table 1 and vertical axis presents the value of GDD-agreement between the original networks and model networks from each model. Lines with different labels correspond to different model networks described in Table 2.

Figure 2 presents GDD agreements between the data and the model networks. We can see that our new model shows an improved fit over all other network models in all of five datasets used. This suggests that our model can successfully fit PPI networks in terms of structural similarity.

### Embedding Quality Comparison between t-LSE and MDS-GEO

Both *t*-LSE and MDS-GEO utilize the connectivity information for fitting PPI networks and output a low-dimensional embedding which can be used to reconstruct the original network by choosing a distance cutoff. Under this scenario, in order to compare the embedding performance of *t*-LSE and MDS-GEO for embedding PPI network, we first learn the following two conditional probability density functions based on the original PPI networks and its embedding space: p(Distance|Interaction) and p(Distance|Non-interaction), where p(Distance|Interaction) describes the distribution of pairwise distances in the embedding space between interacting protein pairs (i.e., form edges in the PPI network) and p(Distance|Non-interaction) describes the distribution of pairwise distances between pairs of proteins which do not interact with each other.

**Figure 3:**
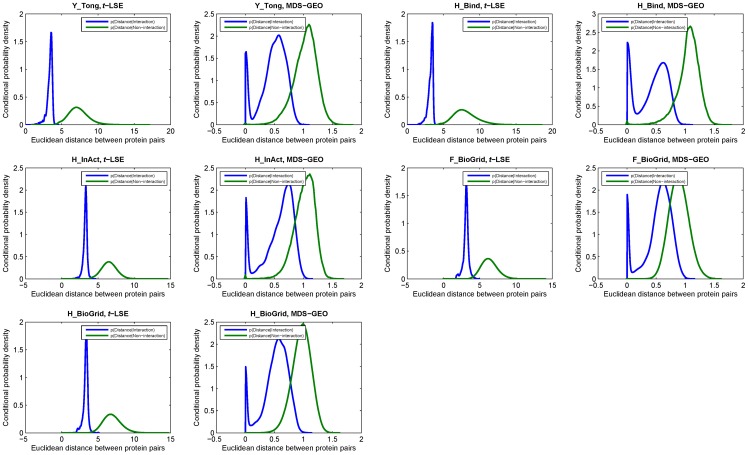
Comparison of the conditional probability density functions learned from embedding the components of 5 networks using *t*-LSE and MDS-GEO.

In Figure 3, we present the probability density functions given by embedding the components of the 5 PPI networks into 20-dimensional Euclidean space using *t*-LSE and MDS-GEO. The *x* axis denotes the distance between pairs of points in the embedding space and the *y* axis denotes the value of conditional probability density function. As can be seen, for all of the five PPI networks, *t*-LSE can achieve a significant improvement over MDS-GEO in terms of the separation between p(Distance|Interaction) and p(Distance|Non-interaction). This means that compared with MDS-GEO, *t-*LSE can better classify the pairs of nodes in the PPI network into interactions and non-interactions based on the similarity between them in the embedding space, the topological structure of the network can thereby be more faithfully preserved.

**Figure 4:**
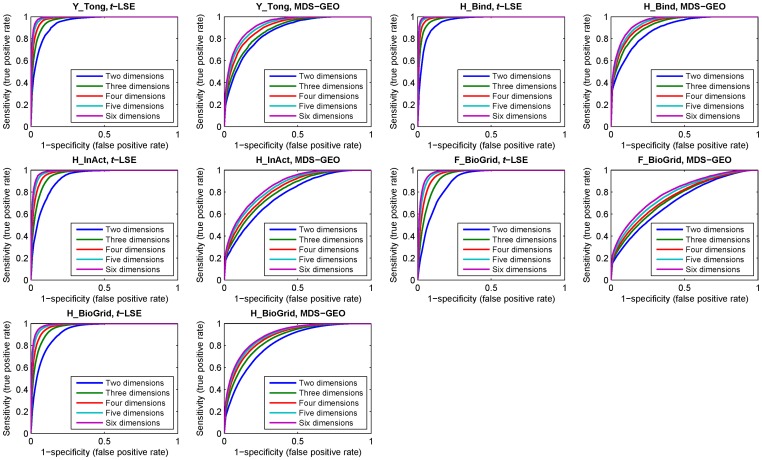
ROC curves comparing the ability of recovering the original 5 networks using *t*-LSE and MDS-GEO with embedding space dimensions of 2 to 6.



As in [15], we further use a ROC curve analysis to evaluate the embedding quality. Figure 4 demonstrates the ROC curves for the five PPI datasets. For each PPI network, the five ROC curves for different embedding space dimensions are constructed by varying the distance threshold from 0 to the maximum distance between the points in the corresponding embedding space. The *x* axis of ROC curve is defined as 1-specificity (or false positive rate) and the *y* axis is defined as sensitivity (or true positive rate). Specificity and sensitivity are two commonly used measures of the performance of a binary classification test, and they are defined as follows.

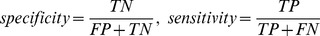



where *TP* (True Positive) is the number of true interacting protein pairs which are predicted to be interacting (the distance between point pair in the embedding space is less than a given distance threshold). *TN* (True Negative) is the number of non-interacting protein pairs that are predicted to be non-interacting (the distance between point pair in embedding space is larger than a given distance threshold). *FP* (False Positive) is the number of non-interacting protein pairs which are predicted to be interacting, and *FN* (False Negative) is the number of interacting protein pairs which are predicted to be non-interacting. It is well known that a ROC curve depicts relative trade-offs between true positive (benefits) and false positive (costs). The best possible ROC curve would contain a point in the upper left corner or coordinate (0, 1) of the ROC space, representing 100% sensitivity (no false negatives) and 100% specificity (no false positives). From Figure 4, we can see that the performance of *t*-LSE is significantly better than MDS-GEO. For example, For Y_Tong network, the sensitivity and specificity of ROC curve of *t*-LSE can reach 97% and 96% respectively when PPI network is embedded into the 6 dimensional space. This corresponds to the false negative rate 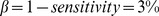 and the false positive rate 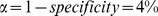. On the other hand, for dimension 6 of the embedding space, the sensitivity and specificity of ROC curve of MDS-GEO can only reach 90% and 80%.

**Figure 5:**
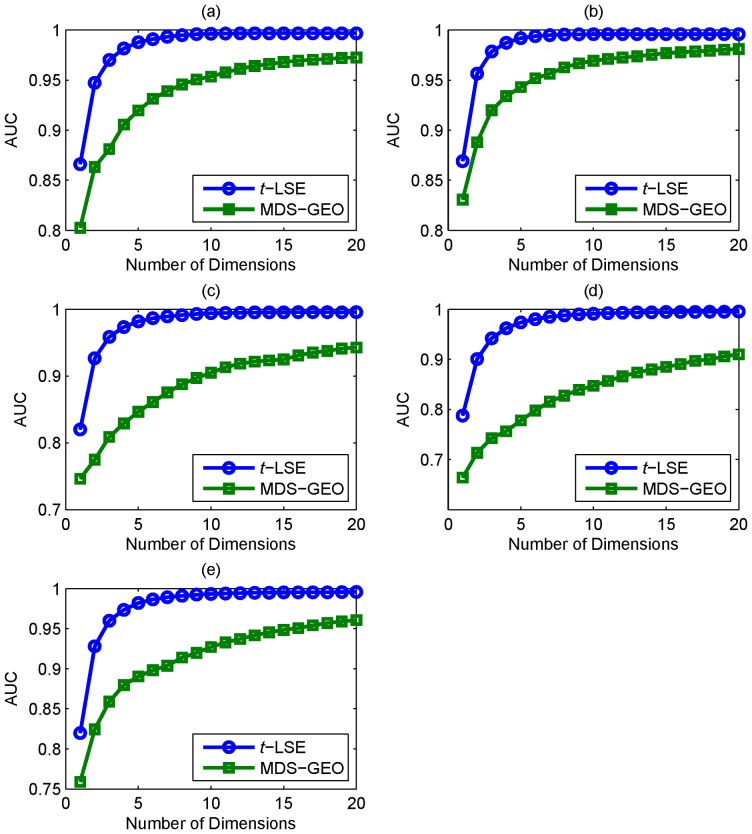
Area under Curve (AUC) comparison. Area under Curve (AUC) comparison measuring the ability of recovering the original PPI networks: (a) Y_Tong, (b) H_Bind, (c) H_InAct, (d) F_BioGrid, (e) H_BioGrid using embedding space dimensions of 1 to 20.

A commonly used assessment metric for ROC curve is the area under the ROC curve (AUC) [15], in Figure 5 we plot the evolving curves of the AUC value as functions of embedding dimensions for *t*-LSE and MDS-GEO. We can see that the AUC value achieved by *t*-LSE is consistently better than MDS-GEO. Figure 5 also shows that for *t*-LSE, the increasing of the embedding space dimension after it exceeds 10 can only slightly improve the AUC. Therefore, the PPI network is well modeled by low dimensional embedding metric space using *t*-LSE.

### Denoising of PPI Networks Using t-LSE and MDS-GEO

The experimental results reported in previous sections confirm that the proposed t-LSE model can accurately preserves the graph topology of the original PPI network. Unfortunately, the noise levels inherent in all current PPI networks are usually very high, our concern is that a well fitting model may be sensitive to noise and have over-fitting problems.

We first investigate the robustness of our model against simulated random noises. More specifically, we randomly remove a subset of connections and randomly insert a subset of connections for the simulation of noisy PPI networks. We generated 20 perturbed networks of each type (corresponding to the percentages of noise), embedded them in the metric space, and computed the AUC using the original unperturbed networks.

**Figure 6:**
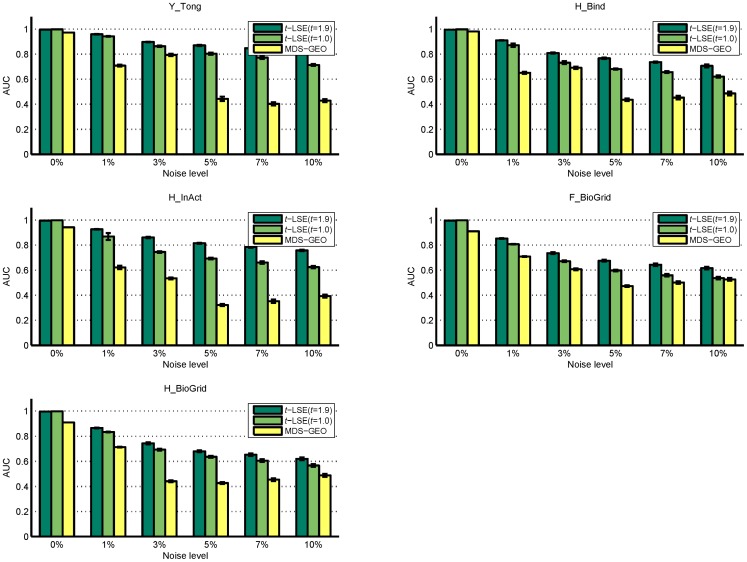
Comparison of AUC values for various methods on 5 networks with different level of noises.

In Figure 6, we plot the means and standards deviations of the AUC achieved by *t*-LSE and MDS-GEO with different levels of noise. Beside the default *t-*logistic loss (*t* = 1.9) used in previous sections, we also report the results of *t*-LSE with *t* = 1.0, where *t*-logistic loss reduces to the standard convex logistic loss function.

For graph embedding algorithm, the AUC can be interpreted as the probability that a connected pair of nodes is given a higher score than a unconnected pair of nodes [27]. A random predictor will give AUC of score 0.5, and the extent to which the AUC exceeds 0.5 reflects how one predicting method is better than random guess. In Figure 6, the comparison of two methods shows that *t*-LSE method is consistently better than that of MDS-GEO in predicting true PPIs indicated by the higher values of AUC. We also notice that after the level of noise exceeds 5%, the performance of MDS-GEO is close to a random predictor, indicated by the AUC value (near 0.5), while *t*-LSE with *t* = 1.9 still performs reasonably. This test confirms that for all three networks, *t*-LSE provides a distinct advantage (especially with *t* = 1.9) over MDS-GEO.

The above experiments prove that our model is robust against random perturbations. However, the real noise properties in PPI data can be different from the simulated random deletions and insertions. Based on the robustness of t-LSE, next we evaluate its performance for identifying unreliable links in the PPI networks.

A number of approaches have been introduced for eliminating unreliable interactions and increasing the reliability of protein interactome. Among them, the network-topology-based methods attracted extensive attention. The representative algorithms include interaction generality (IG) [39], [40], Czekanowski-Dice distance (CD-Dist) [14], and functional similarity weight (FSWeight) [13]. As in *t*-LSE and MDS-GEO, these approaches are promising as they only require the input from the PPI network topology. Unlike *t*-LSE and MDS-GEO however, they are not model-based and the main idea of these methods is to rank the reliability of an interacting protein pair based on the topology of the interactions between the protein pair and their neighbors within a short radius [41].

In the following experiments, CD-Dist, FSWeight and IG are also included for comparison. As in [13], [41], we utilize the degree of functional homogeneity and localization coherence of protein pairs as the measure to evaluate the performance.

It is well known that the strategy of ‘guilt by association’ provides the evidence that interacting proteins are likely to share a common function and cellular localization [42], which means true interacting protein pairs should share at least a common functional role or they should at least be at a common cellular localization if a pair of proteins to be interacting in *vivo*. Since both *t*-LSE and MDS-GEO assume that the distance between two proteins in the embedding space is a monotonically decreasing function of the probability that they interact, it is expected that if we only consider protein pairs with smaller distance in the latent space to be have true positive interaction, the proportion of interacting proteins with functional homogeneity and localization coherence should increase correspondingly.

In the study, the Gene Ontology (GO) based annotations is used to evaluate the functional homogeneity and localization coherence. The GO is one of the most important ontology within the bioinformatics community (see http://www.geneontology.org/). The three organizing principles of Gene Ontology are cellular component, biological process, and molecular function. Here we used the first taxonomies of the GO terms for localization coherence calculation, and the last two taxonomies of the GO terms for functional homogeneity calculation. The GO terms are organized hierarchically into functional subfamilies. Two different GO terms may have a common parent or a common child in the hierarchy. GO terms at high levels may occur in many genes (or proteins), while GO terms at low levels appear in very few proteins. In our experiment, we just choose those GO terms at middle levels. More specifically, we choose the GO terms which occur in at least 30 proteins, but none of its children appears in at least 30 proteins.

**Figure 7:**
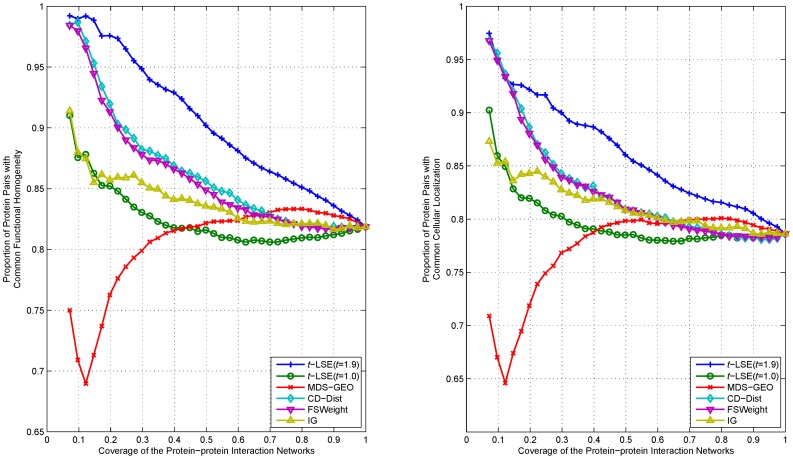
Comparison of various algorithms on Y_Tong network for assessing the reliability of interactions in term of functional homogeneity and localization coherence.

**Figure 8:**
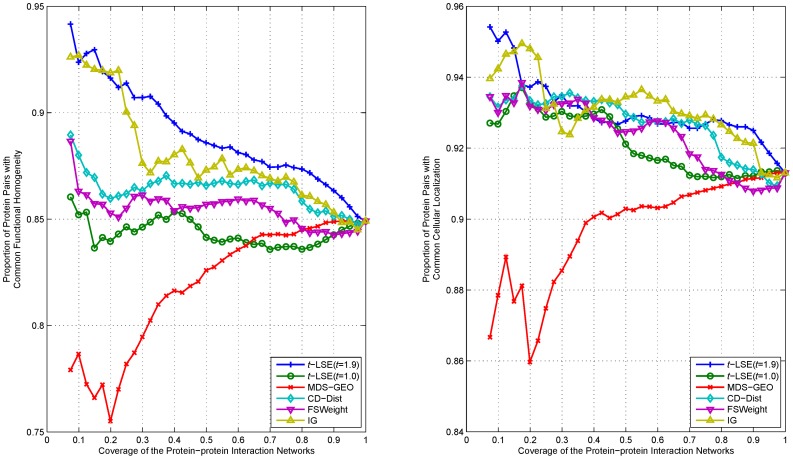
Comparison of various algorithms on H_Bind network for assessing the reliability of interactions in term of functional homogeneity and localization coherence.

**Figure 9:**
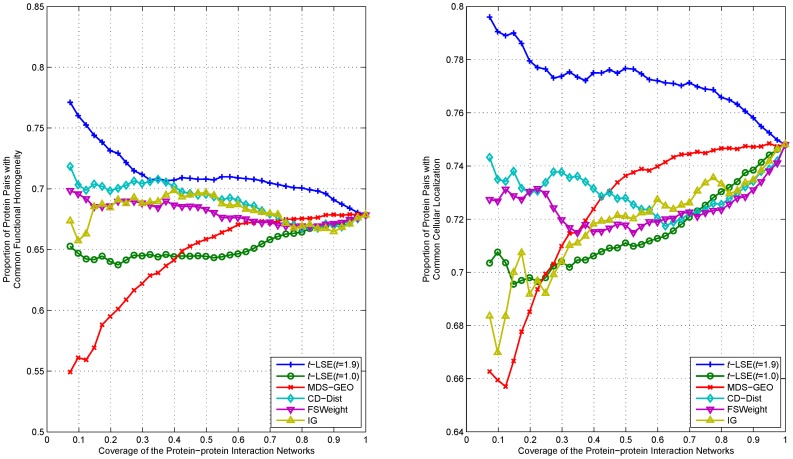
Comparison of various algorithms on H_InAct network for assessing the reliability of interactions in term of functional homogeneity and localization coherence.

**Figure 10:**
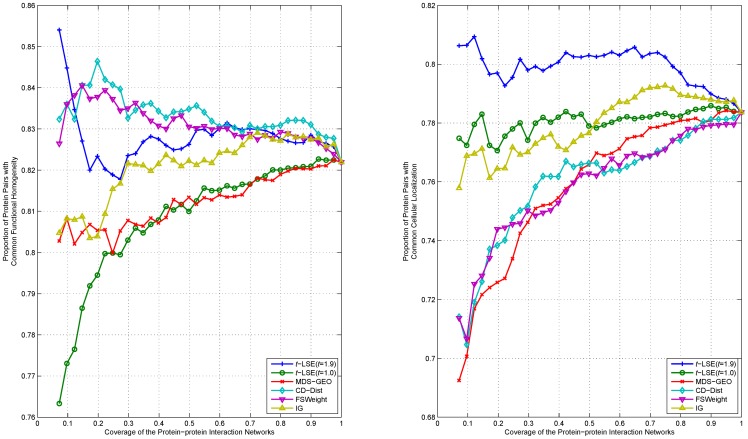
Comparison of various algorithms on F_BioGrid network for assessing the reliability of interactions in term of functional homogeneity and localization coherence.

**Figure 11:**
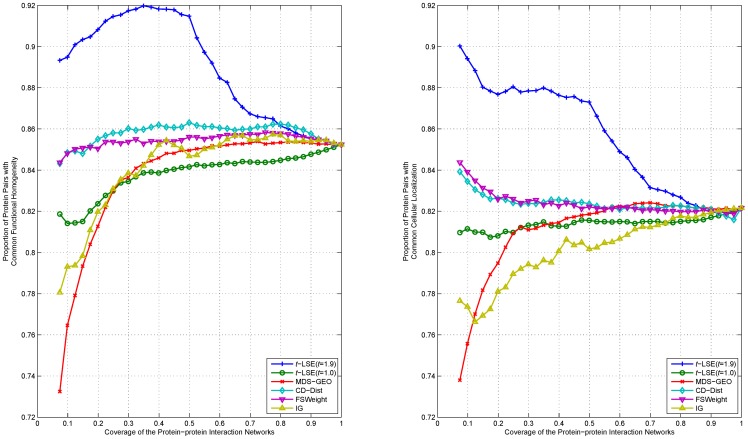
Comparison of various algorithms on H_BioGrid network for assessing the reliability of interactions in term of functional homogeneity and localization coherence.

We rank interactions of proteins according to their distance in the embedding space from the lowest to highest, and measure the functional homogeneity and localization coherence by computing the rate of interacting protein pairs with common functional roles and cellular localization. The experimental results on the three datasets Y_Tong, H_Bind and H_InAct are respectively showed in Figure 7–11. The vertical axis is the proportion of interacting protein pairs which share a common function or cellular localization. The horizontal axis is the coverage of the PPI network comparing the original network.

As can be seen in Figure 7, *t*-LSE with *t* = 1.9 is the best in assessing false positive interactions in the Y_Tong network: as more interactions which were detected as potential false positive interaction were removed from the interactions, the degree of functional homogeneity and localization coherence in the resulting interactome increases at a faster rate than using other methods. 92.9% of the top 40% of interacting protein pairs ranked by *t*-LSE with *t* = 1.9 have a common functional role and 88.7% of them have a common subcellular localization, while the corresponding performance of the best competing method(CD-Dist) are 86.9% and 83.1%.

For H_InAct and H_BioGrid, the conclusions are similar. On the F_BioGrid and H_Bind networks, although *t*-LSE with *t* = 1.9 has no clear advantage over IG and FSWeight, it still achieves comparable performance.

On the whole, *t*-LSE with *t* = 1.9 achieves highly competitive and sometimes even the best performance as compared to the other approaches for increasing the reliability of protein interactomes, which confirms the usefulness of our method.

## Materials and Methods

A PPI network can be naturally represented as a neighborhood graph 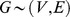, where the set of vertices 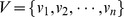 are the proteins, and the set of edges 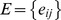 indicate interaction relationships between the proteins. The main idea of our approach is to learn a mapping 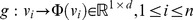 which maps the nodes of *V* into a *d*-dimensional vector space that captures their “semantic similarity”, i.e., we would like the Euclidean distance between node pairs that is known to interact to be smaller than a given threshold 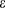 and the distances corresponding to non-interacting pairs to be larger than 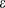, and obtain a probabilistic estimation of whether two nodes interact.

Using the Euclidean distance between 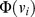
and 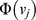, we model the probability 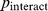 that protein pair 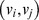 interact, i.e., 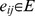, as:
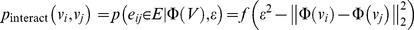
(1)


Correspondingly we model the probability 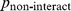 that protein pair 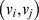 don't interact as
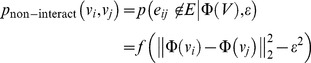
(2)


where 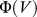 is a 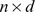 matrix whose *i*-th row is 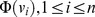, 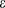 is a bias term and the function 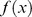 satisfies the properties:

P1. 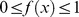;

P2. 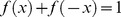;

P3. 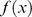 is smooth and increasing.

With properties (P1) and (P2) satisfied we can ensure that 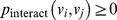, 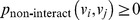 and 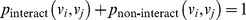; Property (P3) can enforce that a pair of proteins will more likely be assigned an interaction if they are closer to each other in the latent space.

The training objective of *t*-LSE is based on maximum likelihood estimation(MLE), i.e., we minimize the negative log-likelihood function:
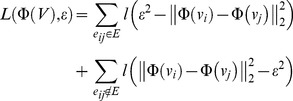
(3)


where 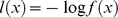.

In order to learn a good embedding of 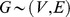 into semantic space, we need to (I) define a robust loss function 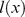 for estimating whether two nodes interact, (II) propose a computationally tractable algorithm for optimizing (3) that can deal with large scale protein networks.

### The *t-*Logistic Loss Function

We first discuss the choice of 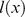. Although the widely used logistic loss 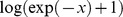 and hinge loss 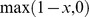 [43] can be used to define 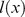, as mentioned earlier, PPI data, as with other high-throughput biological data, contain much noise. It is known that learning algorithms based on convex loss functions such as logistic loss and hinge loss tend to be sensitive to outliers and are not robust in such noisy scenarios [44]. In order to alleviate this problem, many researchers propose to use non-convex loss functions instead [29], [45].

Further inspection of the solution that minimize (3) can give us more insights of the effect of a convex 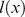: the optimal 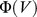 should satisfy that 
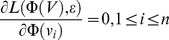
, which yields
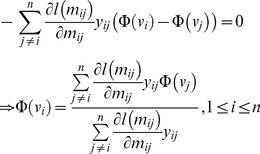
(4)


where 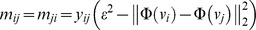, 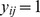 if the protein pair 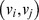 interact and −1 otherwise. In this form, the *i*-th embedded data point 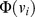 can be regarded as the weighted average of other embedded points, while the value 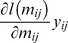 can be thought as the mixing coefficients and indicates the impact of the link 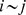 on 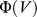. For noisy networks with many false links, we clearly need to control the influence of a specific link, i.e., the absolute value of 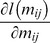. However, if 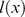 is convex and decreasing, its gradient is an increasing and negative function. This means that false links that tend to cause significant model deviation (i.e., small 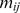) would keep more influence on the optimal solution of (3), which may result in the optimal 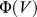 deviating from the original noiseless position and thus deteriorate the performance of the embedding method.

In this paper, we propose using a robust non-convex *t*-logistic loss to limit the impact of noisy links, which has been successfully applied to robust classification tasks and other machine learning applications [28], [46].

The *t*-logistic loss is based on the *t*-exponential family of functions, which is direct generalization of exponential function and for (1<*t*<2) is defined as [47], [48]:
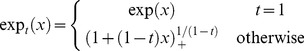
(5)


where 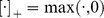.

The inverse function of 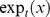 is given by
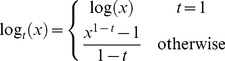
(6)


As in [28], we then define the *t*-logistic loss function 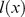 as
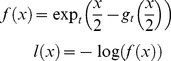
(7)


where 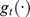 is a function which enforces that 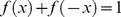.

Although no closed form expression exists for 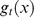 in general, one can compute 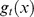 for arbitrary 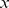 and 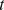 using efficient numerical techniques [47], [48].

It is worthy to note that if 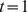, we have 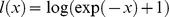
and the *t*-logistic loss function reduces to the standard convex logistic loss [19].

Figure 1 displays the *t*-logistic loss function and its gradient with several different *t*. It is shown that compared to logistic loss, the *t*-logistic loss (especially with larger *t*) increases more slowly when 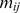 decreases. The gradient 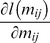 also become a decreasing function as 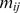 becomes small, which according to our previous analysis, could cap the influence of false links that tend to cause smaller 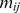.

### The Learning Algorithm for *t-*LSE

The minimization of (3) is a smooth unconstrained optimization problem. In principle, it can be solved using any off-the-shelf solver. However, due to the non-convexity of *t*-logistic loss function, we have noticed that standard methods like gradient descent often lead to poor local minimum during the experiments, thus we adopt an customized alternating projection strategy to minimize 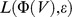 until convergence. More specifically, each time we optimize one parameter, such as 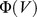, with the other parameters fixed.

The learning of 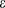 with 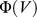 fixed is a simple single variant optimization problem and we solve it using gradient descent method, which works well in practice.

Then we learn 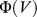 with 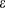 fixed. The partial derivative (4) can be further written as the following compact form:
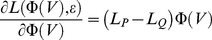
(8)


where 
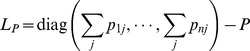
, 
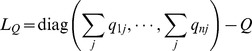
, the matrices 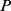 and 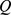 are defined as


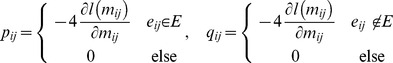
(9)

During the experiments, we have noticed that learning 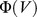 with the standard gradient descent direction (8) is very slow and requires many tiny steps to converge. Letting (8) to zero, we instead investigate several splits in an attempt to identify a fixed point iteration method for *t*-LSE. For instance, we can consider.
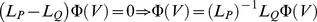
(10)


Although this iteration is not fixed point iteration and does not always converge, it does suggest using a new search direction 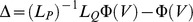 along which we can decrease 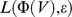 with a line search 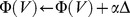 for 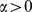. As is proven in Text S1, 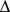 is a descent direction, i.e., the directional derivative of the search direction always remains negative. Hence, as a result of Zoutendijk's theorem, we are guaranteed to converge to a local optimum of 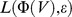 if we use the search direction in combination with a line-search that satisfies the Wolfe conditions [49].

It is worthy to note that we can use an off-the-shelf linear system solver to compute 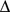 and the matrix inversion 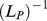 does not need to be calculated explicitly. It is also easy to verify that the cardinality of the matrix 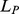 is 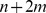, since PPI networks are typically very sparse, with average degree of 7 or less [12], 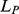 is also very sparse. Therefore we use the sparse linear system solver LSQR [50] to compute 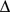, which is much more efficient than dense linear system solvers like Cholesky decomposition based methods [51].

## Supporting Information

Text S1 **The implementation details and convergence results of** ***t*** **-LSE.**
(PDF)
