# Supplementary material for: t-LSE: A Novel Robust Geometric Approach for Modeling Protein-Protein Interaction Networks
Source: PLoS One. 2013 Apr 1;8(4):e58368. doi: 10.1371/journal.pone.0058368 (PMC3613363; doi:10.1371/journal.pone.0058368)
Supplement: Text S1 — The implementation details and convergence results of t -LSE. (PDF) [file pone.0058368.s001.pdf]

Convergence Result of *t*-LSE

The gradient of $L\left(\Phi(V),\varepsilon\right)$ can be written as the following compact form:

$$\frac{\partial L\left(\Phi(V),\varepsilon\right)}{\partial \Phi(V)} = \left(L_P - L_Q\right)\Phi(V)$$

where L_P and L_Q represent

$$L_P = \mathrm{diag}\left(\sum_j p_{1j}, \sum_j p_{2j}, \cdots, \sum_j p_{nj}\right) - P\ ,\ \ L_Q = \mathrm{diag}\left(\sum_j q_{1j}, \sum_j q_{2j}, \cdots, \sum_j q_{nj}\right) - Q$$

The matrices P and Q are defined as

$$p_{ij} = \begin{cases} -4\lambda_{ij} \dfrac{\partial l_{ij}\left(\Phi(V),\varepsilon\right)}{\partial m_{ij}} & e_{ij} \in E \\ 0 & \text{else} \end{cases},\ \ q_{ij} = \begin{cases} 4\lambda_{ij} \dfrac{\partial l_{ij}\left(\Phi(V),\varepsilon\right)}{\partial m_{ij}} & e_{ij} \notin E \\ 0 & \text{else} \end{cases}$$

Here we prove that the search direction $\Delta = \left(L_P\right)^{-1} L_Q \Phi(V) - \Phi(V)$ is a descent direction. The inner-product between Δ and $\left(L_P - L_Q\right)\Phi(V)$ is

$$\begin{aligned} \left\langle \Delta, \left(L_P - L_Q\right)\Phi(V)\right\rangle &= \mathrm{trace}\left(\Delta^T \left(L_P - L_Q\right)\Phi(V)\right) \\ &= -\mathrm{trace}\left(\Delta^T L_P \Delta\right) \end{aligned}$$

Thus we only need to prove that L_P is positive semi-definite. It is easy to verify that L_P is symmetric, and for any $a = \left[a_1, a_2, \cdots, a_n\right]^T \in \mathbb{R}^n$,

$$a^T L_P a = -4\sum_{i=1}^{n} \sum_{j=i+1}^{n} \lambda_{ij} \frac{\partial l_{ij}\left(\Phi(V),\varepsilon\right)}{\partial m_{ij}} \left(a_i - a_j\right)^2$$

Since $l_{ij}\left(\Phi(V),\varepsilon\right)$ is a decreasing function of m_{ij}, λ_{ij} is positive, we have $a^T L_P a \geq 0$. Thus we have L_P is positive semi-definite. Hence, as a result of Zoutendijk' s theorem [38], we are guaranteed to converge to a local optimum of $L\left(\Phi(V),\varepsilon\right)$ if we use the search direction in combination with a line-search that satisfies the Wolfe conditions, i.e., a line-search step that simultaneous satisfies the Armijo condition and the curvature condition.
